# The effect of auditors’ digital literacy on reduced audit quality behavior: A chain mediation model and the moderating role of prosocial behavior

**DOI:** 10.1371/journal.pone.0350835

**Published:** 2026-07-02

**Authors:** Maobao Yang, Lei Guo, Gaofei Ren, Qiuhong Wang, Yi Liu

**Affiliations:** 1 School of Management, Jiujiang University, Jiujiang, China; 2 Jiangxi Yangtze River Economic Zone Research Institute, Jiujiang, China; Liverpool John Moores University, UNITED KINGDOM OF GREAT BRITAIN AND NORTHERN IRELAND

## Abstract

Reduced audit quality behavior is prevalent in audit practice and poses a serious threat to overall audit quality. With the increasing adoption of digital auditing, auditors’ digital literacy has become essential not only for adapting to the modern audit environment but also for improving audit quality and curbing inappropriate audit practices. This study examines the effect of auditors’ digital literacy on reduced audit quality behavior by introducing digital self-efficacy and task performance as mediating variables and constructing a chain mediation model, while also exploring the moderating role of prosocial behavior. Empirical analysis based on 480 valid questionnaires revealed that digital literacy negatively influences reduced audit quality behavior. Digital self-efficacy and task performance partially mediate this relationship. Additionally, digital self-efficacy positively enhances task performance, resulting in a chain mediating effect. Prosocial behavior moderates these relationships, significantly affecting the mediating roles of digital self-efficacy and task performance. Accordingly, fostering a digitally enabled audit environment that enhances auditors’ analytical efficiency, judgment accuracy, and procedural compliance requires auditors to effectively leverage digital technologies to mitigate audit quality-threatening behavior.

## 1. Introduction

Audit quality is fundamental to the auditing profession, underpinning both its sustainability and the effective execution of audit functions [1]. Auditors may engage in reduced audit quality behaviors that, although often covert, pose significant threats to audit quality and may result in adverse consequences [2]. With the rapid expansion of digital technologies, the advancement and digital transformation of auditing practices have become inevitable. These developments facilitate the integration of digital technologies into audit practice, thereby enhancing efficiency and audit quality [3]. Accordingly, examining how auditors’ digital literacy mitigates reduced audit quality behavior is of significant practical importance, as it provides actionable insights for designing targeted training programs, optimizing audit processes, and improving audit quality in digitalized environments.

Existing research identifies reduced audit quality behavior as a critical yet often unobservable phenomenon that can substantially undermine audit effectiveness [4]. Such behavior refers to actions by auditors that result in insufficient or questionable audit evidence, thereby compromising audit rigor and professional credibility [5]. Prior studies have examined its antecedents and mitigation mechanisms, highlighting factors such as time pressure, cost constraints, and information asymmetry. Under such pressures, auditors may truncate procedures, prematurely terminate required steps, conduct superficial reviews, or accept vague client explanations, thereby increasing audit risk and weakening audit quality [6,7].

Building on this literature, recent studies increasingly emphasize the role of technological adaptability in audit effectiveness. Technological innovation has been shown to mediate the relationship between adaptability and improved audit outcomes [8]. Digital technologies support the modernization of audit practice by enhancing efficiency, analytical depth, and professional standards [9]. Moreover, technology-driven approaches help alleviate tensions between limited resources and increasing audit complexity, thereby improving audit efficiency and effectiveness [10].

Drawing on human capital theory, this study conceptualizes auditors’ digital literacy as a specialized form of human capital that enhances their capacity to understand and apply digital technologies in audit work, thereby optimizing processes and improving audit quality [11]. Although enterprise digitalization may increase audit effort and complexity, improved outcomes are not automatic. In the digital era, enhancing auditors’ digital literacy has become a critical imperative and constitutes the central focus of this study [12].

Improving audit quality requires enhancing auditors’ self-efficacy, efficiency, and overall performance [13]. Expertise in information technology(IT) enhances certified public accountants’ (CPAs’) digital self-efficacy, reinforcing their confidence in performing audit tasks effectively [14]. Innovative technologies strengthen system robustness, improve execution effectiveness, and optimize the processing of audit information [15]. Accordingly, digital literacy may indirectly reduce audit quality-threatening behavior by enhancing digital self-efficacy and task performance; therefore, these constructs are modeled as mediating variables.

The chain mediating relationship between digital self-efficacy and task performance is grounded in social cognitive theory, which posits that self-efficacy is a central cognitive mechanism linking individual capabilities to behavioral outcomes [16]. Digital self-efficacy influences task performance by shaping goal-setting, effort intensity, persistence under pressure, and resilience when facing complex digital audit tasks [17]. Auditors with high digital self-efficacy are more likely to adopt proactive problem-solving strategies, invest sustained effort in data analysis, and persist in completing demanding audit procedures, thereby achieving superior performance [11]. In this context, digital literacy enhances perceived competence with digital tools, which in turn translates into higher task performance [18]. This sequential process constitutes a coherent chain mediation mechanism through which digital literacy ultimately reduces reduced audit quality behavior via both cognitive and behavioral pathways.

## 2. Theoretical background and research hypotheses

### 2.1. Digital literacy and reduced audit quality behavior

Digital literacy is commonly defined as the ability to use digital technologies to create, evaluate, and share information [19]. In the auditing context, auditors’ digital literacy refers to an integrated capability comprising the knowledge, skills, and attitudes required to perform audit tasks effectively in digital environments [20]. As auditing increasingly relies on technologies such as big data analytics and artificial intelligence, insufficient digital competence may lead to inefficiencies and judgment inaccuracies, thereby undermining audit quality [3].

Auditors with high digital literacy are better able to process large volumes of complex data, identify anomalies and risk signals, and apply analytical tools to support evidence-based judgment [12]. Familiarity with audit software and data analytics platforms enhances audit efficiency, reduces human error, and supports the automation of routine procedures [9]. These capabilities enable auditors to allocate effort more effectively, reduce unnecessary procedures, and focus on high-risk areas, thereby reducing the likelihood of reduced audit quality behavior [21]. Accordingly, the following hypothesis is proposed:

H1: Digital literacy has a negative impact on reduced audit quality behavior.

### 2.2. Digital self-efficacy and task performance

Digital self-efficacy refers to individuals’ confidence in their ability to use digital technologies to accomplish work tasks successfully [16]. Employees with high digital self-efficacy exhibit greater adaptability and confidence when responding to challenges in digital environments [14]. Digital literacy enhances this confidence by enabling individuals to acquire, evaluate, and apply digital information effectively, thereby strengthening task-related problem-solving capability [22].

Task performance reflects work outcomes resulting from the execution of core job duties and serves as a direct indicator of employee effectiveness [23]. From a person-task-technology fit perspective, digital skills enable employees to align task demands with technological resources, improving efficiency and accuracy [24]. In auditing, digital tools such as data analytics and artificial intelligence facilitate faster evidence processing, more accurate risk assessments, and more effective audit planning, thereby enhancing task performance [25]. Accordingly, auditors with stronger digital literacy are more likely to exhibit higher digital self-efficacy and demonstrate superior task performance. The following hypotheses are proposed:

H2: Digital literacy has a positive impact on digital self-efficacy.

H3: Digital literacy has a positive impact on task performance.

### 2.3. Digital self-efficacy, task performance and reduced audit quality behavior

Auditors’ attitudes and behavioral tendencies play a critical role in shaping audit outcomes [2]. Prior research indicates that auditors with higher digital self-efficacy are more likely to approach complex tasks proactively, optimize audit processes, and maintain professional diligence, even under pressure [13]. Confidence in using digital tools enables auditors to apply advanced analytical techniques, improve audit accuracy, and reduce reliance on heuristic judgments that may compromise audit quality [9].

Task performance is also directly related to audit quality. Low efficiency and excessive workload may prompt auditors to truncate procedures or engage in superficial reviews, increasing the likelihood of reduced audit quality behavior [6]. By contrast, higher task performance allows auditors to allocate sufficient time to critical audit areas, conduct deeper analyses, and reduce errors and omissions [7]. Accordingly, the following hypotheses are proposed:

H4: Digital self-efficacy has a negative impact on reduced audit quality behavior.

H5: Task performance has a negative impact on reduced audit quality behavior.

### 2.4. The mediating roles of digital self-efficacy and task performance

Auditors’ effective use of digital technologies enhances their digital self-efficacy by reinforcing perceived control over technology-enabled audit tasks. From a social cognitive perspective, repeated mastery experiences strengthen auditors’ confidence in performing complex audit activities [25]. Heightened digital self-efficacy facilitates sustained effort, persistence, and proactive problem solving, which are critical antecedents of task performance [26]. While digital literacy provides the technical capability to process and interpret complex audit data, digital self-efficacy determines whether this capability is translated into effective task execution [9].

Effective utilization of digital technologies improves audit efficiency through faster information processing, broader audit coverage, and more precise risk identification [27]. However, these performance gains materialize only when auditors possess sufficient digital self-efficacy to actively engage with advanced technologies [3]. Consequently, digital literacy influences audit quality through a sequential psychological-behavioral mechanism, whereby enhanced digital self-efficacy improves task performance, which ultimately reduces reduced audit quality behavior [28]. This logic supports a chain mediation model:

H6: Digital self-efficacy mediates the relationship between digital literacy and reduced audit quality behavior.

H7: Task performance mediates the relationship between digital literacy and reduced audit quality behavior.

H8: Digital self-efficacy and task performance jointly form a chain mediating mechanism between digital literacy and reduced audit quality behavior.

### 2.5. The moderating effect of prosocial behavior

Prosocial behavior refers to voluntary, other-oriented actions that facilitate cooperation, knowledge sharing, and mutual support in organizational contexts [29]. In audit teams, such behavior promotes information exchange and collaborative problem solving, which are particularly important in digitally intensive environments [30].

Prosocial behavior strengthens the effect of digital literacy by creating a supportive social environment. Drawing on social cognitive theory, such behavior enhances perceived social support, reduces uncertainty associated with adopting new technologies, and increases confidence in digital capabilities [18]. Through mechanisms such as peer assistance, mentoring, and knowledge sharing, prosocial behavior reinforces mastery experiences and accelerates the transformation of digital literacy into digital self-efficacy.

In turn, supportive social interactions facilitate the translation of digital self-efficacy into task performance [31]. Auditors in prosocial environments are more likely to engage deeply with digital tools, leading to improved task execution and reduced audit quality-threatening behavior [32]. Accordingly, prosocial behavior not only moderates the direct relationship between digital literacy and reduced audit quality behavior but also strengthens the indirect effects operating through digital self-efficacy and task performance.

H9: Prosocial behavior moderates the relationship between digital literacy and reduced audit quality behavior.

H10: Prosocial behavior moderates the relationship between digital literacy and digital self-efficacy.

H11: Prosocial behavior moderates the relationship between digital literacy and task performance.

H12: Prosocial behavior moderates the mediating effect of digital self-efficacy in the relationship between digital literacy and reduced audit quality behavior.

H13: Prosocial behavior moderates the mediating effect of task performance in the relationship between digital literacy and reduced audit quality behavior.

**Fig 1:**
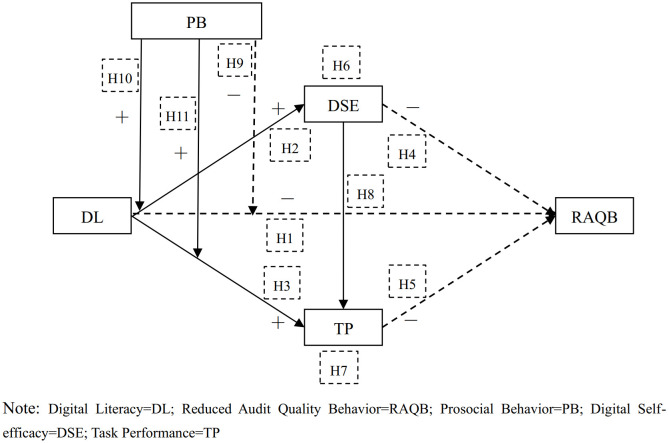
Theoretical model.

Building on the above discussion, Fig 1 presents the proposed theoretical model, illustrating the hypothesized relationships among digital literacy, digital self-efficacy, task performance, prosocial behavior, and reduced audit quality behavior.

## 3. Methods

### 3.1. Sample selection and data collection

This study administered a questionnaire to practicing auditors using a mixed online and offline distribution strategy, aiming to obtain a heterogeneous sample across gender, age groups, organizational types, and professional experience. An established set of scales was adopted and refined to reflect the research context and target population, yielding the final survey instrument. The questionnaire comprises five sections: (1) demographic and professional background, (2) prosocial behavior, (3) digital self-efficacy, (4) task performance, and (5) reduced audit quality behavior, totaling 27 items, with 6 items capturing respondents’ demographic characteristics.

The study protocol was reviewed and approved by the Ethics Committee of the School of Management, Jiujiang University. Between April and July 2025, data were collected from certified public accounting firms using both online and paper-and-pencil questionnaires. An introductory statement at the beginning of the questionnaire explained the study’s purpose, assured anonymity and confidentiality, stated that participation was voluntary, and described data handling procedures. Written informed consent was obtained from all participants: paper-based respondents signed a written consent form, while online respondents provided electronic consent by clicking an “I agree” button. No personally identifiable information was collected. This study did not involve minors.

A total of 523 questionnaires were distributed. After data screening, 43 unqualified or invalid questionnaires were removed. Finally, 480 valid questionnaires were obtained, corresponding to an effective response rate of 91.78%. Sample characteristics based on the 480 valid responses are reported in Table 1. Table 1 summarizes the demographic and professional characteristics of the sample, providing an overview of respondents’ gender, age, education level, organizational type, and work experience. Demonstrating substantial variation in respondents’ age, education, job level, organizational type, and work experience.This sampling strategy was adopted due to the practical difficulty of obtaining a comprehensive sampling frame of auditors and the restricted access to audit professionals, which is common in auditing research. Although the use of convenience sampling may limit the statistical generalizability of the findings, the relatively large sample size and diversity of respondent characteristics enhance the robustness and external relevance of the results within comparable audit contexts. Accordingly, the findings should be interpreted as analytically generalizable to auditors operating in similar institutional and professional environments rather than universally generalizable to all auditors.

**Table 1:** Statistical characteristics.

Variable		N	PCT(%)	Variable		N	PCT(%)
Gender	Male	332	69.167	OrgType	Private	386	80.417
	Female	148	30.833		State-owned	94	19.583
Age	Under 30	79	16.458	WorkExp	Under 3	72	15.000
	31 ～ 40	168	35.000		4 ～ 10	158	32.917
	41 ～ 50	148	30.833		11 ～ 15	146	30.417
	Over 51	85	17.708		Over 16	104	21.667
Level	Junior	85	17.708	Education	Associate & below	139	28.958
	Intermediate	234	48.750		Bachelor	272	56.667
	Senior	161	33.542		Graduate	69	14.375

### 3.2. Variable measurement

All measurement scales were adapted through a structured procedure combining prior literature, pilot testing, and expert evaluation to ensure contextual relevance and psychometric rigor. All measurement scales were adapted following a standardized translation and back-translation procedure to ensure linguistic and conceptual equivalence. Two bilingual scholars independently translated the original English items into Chinese, after which a separate bilingual expert back-translated the items into English. Discrepancies were discussed and resolved through consensus. A pilot survey was subsequently conducted to assess item clarity, contextual appropriateness, and content validity. Prior to formal data collection, a pilot study was conducted to assess item clarity, contextual appropriateness, and preliminary psychometric properties. Based on pilot feedback and statistical diagnostics, minor wording adjustments were made, and redundant or contextually inappropriate items were removed. All perceptual constructs were measured using five-point Likert scales. Unless otherwise specified, scale anchors ranged from 1 (strongly disagree) to 5 (strongly agree).

1. Digital Literacy. Digital literacy was measured using a refined version of Durmuş’s [19] 10-item scale. A pilot study was conducted to evaluate item discrimination and contextual suitability. Items emphasizing general ICT use for learning were excluded due to low item-total correlations and limited relevance to auditors’ professional tasks. The final five items demonstrated satisfactory discrimination and content validity, capturing auditors’ capability to acquire, apply, and continuously update digital technologies in audit practice.
2. Reduced Audit Quality Behavior. Reduced audit quality behavior was measured using a 6-item scale based on Zhao et al. [4], which itself builds on Smith and Emerson [33]. To better reflect contemporary audit practice, one additional item addressing time pressure arising from strict deadlines was incorporated. Respondents indicated how frequently they engaged in each behavior over the past year using a frequency-based scale ranging from 1 (never) to 5 (nearly always). Pilot testing confirmed acceptable reliability and item discrimination.
3. Digital Self-Efficacy. Digital self-efficacy was assessed using a validated 3-item scale developed by Yongyue and Lihua [17], drawing on Ulfert-Blank and Schmidt [16]. The scale measures auditors’ confidence in effectively using digital technologies to perform work tasks. Pilot analysis showed strong internal consistency and clear factor structure.
4. Task Performance.Task performance was measured using a 3-item scale developed by Yongwei et al. [34], based on Lam et al. [35] and Çalışkan and Köroğlu [36]. The scale evaluates respondents’ perceived effectiveness in fulfilling core job responsibilities. Pilot results indicated satisfactory reliability and construct validity.
5. Prosocial Behavior. Prosocial behavior was assessed using an adapted version of the auditor-to-client prosocial behavior scale developed by Douthit et al. [32]. Given that the original scale emphasized auditor-client interactions, one item focusing primarily on client-oriented altruism was removed. The remaining items were reworded to reflect prosocial behaviors within audit teams, such as assisting colleagues, sharing expertise, and providing technical support. These modifications were guided by content relevance and expert feedback to ensure alignment with the study’s conceptual definition of prosocial behavior.
6. Control Variables. Consistent with prior audit research [31], gender, age, education level, and work experience were included as control variables. Job level and organization type were also controlled to account for differences in supervisory responsibilities and organizational environments.

Based on human capital theory and prior audit behavior research, several demographic and professional characteristics were included as control variables in the theoretical model [37]. Gender, age, education, and work experience reflect accumulated human capital, cognitive maturity, and professional exposure, all of which may influence auditors’ digital literacy, self-efficacy, and behavioral responses [11]. Job level determines task complexity, autonomy, and decision-making authority, which may affect task performance and audit quality behavior. Organization type shapes access to digital resources, training opportunities, and institutional norms, thereby influencing auditors’ digital competence and behavior [18]. Controlling for these variables ensures that the observed relationships among digital literacy, digital self-efficacy, task performance, and reduced audit quality behavior are not confounded by structural or demographic differences, thereby strengthening the alignment between the theoretical framework and empirical design.

This study relies primarily on self-reported measures for task performance and reduced audit quality behavior due to the inherent confidentiality and accessibility constraints associated with objective audit data, such as internal quality inspection records or project-level performance metrics. Task performance therefore captures auditors’ perceived effectiveness in achieving core job responsibilities, which has been widely adopted in prior audit and organizational research. Reduced audit quality behavior was measured using self-reported frequency-based items, a standard approach in audit research when objective violation data are unavailable. To mitigate potential social desirability bias, respondents were assured of anonymity and confidentiality, participation was voluntary, and no identifying information was collected. In addition, reverse-coded items were included, and common method bias was assessed using Harman’s single-factor test, which indicated no serious concern. Prior empirical studies suggest that, under such controls, self-reported measures provide valid and reliable proxies for auditors’ behavioral tendencies.

### 3.3. Common method bias and reliability and validity test

Given that all variables were collected using a self-reported questionnaire, potential common method bias (CMB) was carefully addressed through both procedural and statistical remedies. Procedurally, respondent anonymity and confidentiality were assured, participation was voluntary, and the questionnaire was structured to reduce evaluation apprehension and consistency motives by separating predictor and criterion variables across different sections. Statistically, Harman’s single-factor test was conducted as an initial diagnostic. An unrotated exploratory factor analysis including all substantive measurement items (excluding control variables) revealed five distinct factors with eigenvalues greater than 1. The largest factor accounted for 31.193% of the total variance, which is below the commonly accepted threshold of 40%. This result suggests that no single latent factor dominated the covariance structure, indicating that common method bias was unlikely to substantially influence the findings [38]. In addition, multicollinearity diagnostics were performed using variance inflation factors (VIFs). All VIF values were below the conservative threshold of 3.3, suggesting that multicollinearity does not threaten the stability of the regression estimates. Prior to hypothesis testing, data normality and potential outliers were assessed through skewness and kurtosis statistics, as well as examinations of standardized residuals and leverage values. All diagnostic results fell within acceptable ranges, indicating no severe violations of statistical assumptions [39].

To further establish measurement reliability, convergent validity, discriminant validity, and overall model robustness, confirmatory factor analysis (CFA) was performed using SPSSAU. As reported in Table 2, Table 2 presents the results of the confirmatory factor analysis, including standardized factor loadings, composite reliability, average variance extracted, and discriminant validity indicators, thereby confirming the reliability and validity of the measurement model. All standardized factor loadings exceeded the recommended threshold of 0.70 and were statistically significant. The average variance extracted (AVE) values ranged from 0.630 to 0.732, exceeding the minimum criterion of 0.50, while composite reliability (CR) values ranged from 0.857 to 0.922, surpassing the recommended threshold of 0.80. Cronbach’s alpha coefficients for all constructs were above 0.80, indicating strong internal consistency. Sampling adequacy and factorability were assessed using the Kaiser-Meyer-Olkin (KMO) measure and Bartlett’s test of sphericity. The KMO value of 0.875 exceeded the recommended minimum of 0.80, and Bartlett’s test was statistically significant, confirming the suitability of the data for factor analysis.

**Table 2:** Reliability and validity（CFA).

Variable	SFL	CR	AVE	α	KMO	1	2	3	4	5
DL	0.817 ～ 0.854	0.922	0.704	0.910	0.875	0.839				
DSE	0.760 ～ 0.844	0.857	0.667	0.813		0.299	0.817			
TP	0.842 ～ 0.876	0.891	0.732	0.890		0.319	0.359	0.856		
PB	0.812 ～ 0.868	0.905	0.704	0.870		0.058	0.211	0.196	0.704	
RAQB	0.768 ～ 0.835	0.911	0.630	0.901		−0.286	−0.320	−0.397	−0.223	0.794

Note: Digital Literacy = DL; Reduced Audit Quality Behavior = RAQB; Prosocial Behavior = PB; Digital Self-efficacy = DSE; Task Performance = TP

Confirmatory factor analysis indicated an acceptable overall model fit across multiple absolute, incremental, and parsimonious indices: χ²/df = 2.137, GFI = 0.933, IFI = 0.967, NFI = 0.940, CFI = 0.967, and TLI = 0.959, all exceeding recommended thresholds. Parsimony-adjusted indices (PGFI = 0.683, PNFI = 0.757, PCFI = 0.778) were also within acceptable ranges. Error-based indices (RMR = 0.031, RMSEA = 0.049, SRMR = 0.032) indicated a good fit between the measurement model and the observed data. Discriminant validity was assessed using the Fornell-Larcker criterion. The square roots of the AVEs for each construct exceeded all corresponding inter-construct correlation coefficients, providing strong evidence of adequate discriminant validity [39]. Collectively, these results confirm that the measurement model demonstrates satisfactory reliability, validity, and robustness.

## 4. Empirical analysis and results

### 4.1. Correlation analysis

This study used SPSSAU to perform the correlation analysis. Table 3 reports descriptive statistics and Spearman correlation coefficients among the study variables, offering preliminary evidence of the hypothesized relationships. As shown in Table 3, the Spearman correlation coefficient was applied to assess relationship strength. Digital literacy (DL) was significantly correlated with digital self-efficacy (DSE), task performance (TP), and reduced audit quality behavior (RAQB). The correlation coefficients were 0.299, 0.266, and −0.253, respectively, all significant at the 0.01 level. These results indicate that DL is significantly associated with digital self-efficacy, task performance, and reduced audit quality behavior [40]. By contrast, the correlation between DL and prosocial behavior (PB) was not significant (p > 0.05), suggesting no significant association between the two variables [41].

**Table 3:** Correlation analysis.

Variable	M	SD	DL	DSE	TP	PB	RAQB
DL	3.509	0.873	1				
DSE	3.251	0.838	0.299^**^	1			
TP	3.349	0.976	0.266^**^	0.346^**^	1		
PB	3.454	0.706	0.088	0.208^**^	0.158^**^	1	
RAQB	2.576	0.841	−0.253^**^	−0.305^**^	−0.402^**^	−0.245^**^	1

Note: *p < 0.05 **p < 0.01; Digital Literacy = DL; Reduced Audit Quality Behavior = RAQB; Prosocial Behavior = PB; Digital Self-efficacy = DSE; Task Performance = TP

### 4.2. Direct effect test

Table 4 reports the regression results for the direct, mediating, and moderating effect tests, forming the empirical basis for hypothesis testing, including both unstandardized and standardized coefficients. Reporting standardized coefficients facilitates comparison of effect magnitudes across predictors and enhances the interpretability of the empirical findings. The results indicate that digital literacy exhibits a statistically significant negative effect on audit quality-threatening behavior, with standardized coefficients demonstrating substantive explanatory power. Furthermore, digital self-efficacy and task performance both show significant mediating effects, and the consistency of standardized coefficients across models indicates robust effect patterns. Overall, the results provide strong empirical support for the proposed theoretical framework and underscore the relative importance of task performance as the primary mediating mechanism.

**Table 4:** Regression results analysis.

Variable	RAQB				DSE		TP		
	M1	M2	M3	M4	M5	M6	M7	M8	M9
Gender	−0.075	−0.095	−0.078	−0.085	−0.038	−0.028	0.010	0.032	0.019
OrgType	0.050	0.036	0.054	0.045	−0.024	−0.019	0.023	0.038	0.027
Age	−0.221	−0.178	−0.158	−0.133	0.084	0.010	0.126	0.077	0.058
WorkExp	0.251	0.214	0.198	0.147	−0.085	0.004	−0.111	−0.070	−0.030
Level	−0.062	−0.052	−0.032	−0.040	0.008	−0.012	0.059	0.047	0.041
Education	0.101	0.092	0.078	0.051	0.005	0.046	−0.038	−0.028	0.000
DL	−0.289^***^			−0.254^***^	0.301^***^	0.275^***^	0.323^***^		0.299^***^
DSE		−0.323^***^						0.361^***^	
TP			−0.396^***^						
PB				−0.175^***^		0.179^***^			0.164^***^
PB*DL				−0.215^***^		0.132^**^			0.120^**^
R^2^	0.099	0.120	0.172	0.183	0.092	0.146	0.108	0.135	0.153
Adj. R^2^	0.085	0.107	0.159	0.167	0.079	0.130	0.095	0.122	0.137
DW	2.110	2.038	2.141	2.122	2.005	1.959	2.092	2.076	2.083

Note: *p < 0.05 **p < 0.01; Digital Literacy = DL; Reduced Audit Quality Behavior = RAQB; Prosocial Behavior = PB; Digital Self-efficacy = DSE; Task Performance = TP

This study employed SPSSAU to examine the direct, mediating, and moderating effects using hierarchical regression analysis, a widely adopted approach in behavioral accounting and audit research [5,42]. As shown in Table 4, Model 1 (M1) indicated a significant negative association between digital literacy (DL) and reduced audit quality behavior (RAQB) (β = −0.289, p < .001), consistent with prior evidence that enhanced digital competence mitigates dysfunctional audit behavior [43,44]. Model 5 (M5) showed a significant positive association between DL and digital self-efficacy (DSE) (β = 0.301, p < .001), supporting social cognitive theory, which posits that skill mastery strengthens individuals’ self-efficacy beliefs [41]. Model 7 (M7) further revealed a significant positive association between DL and task performance (TP) (β = 0.323, p < .001), aligning with empirical research indicating that digital capabilities enhance task efficiency and decision quality in audit contexts [5].

Model 2 (M2) demonstrated a significant negative association between DSE and RAQB (β = −0.323, p < .001), suggesting that auditors with higher digital self-efficacy are less inclined to engage in behaviors that threaten audit quality [42]. Similarly, Model 3 (M3) showed a significant negative association between TP and RAQB (β = −0.396, p < .001), corroborating prior findings that higher task performance alleviates time pressure and reduces the likelihood of procedural shortcuts during audits [41]. Overall, the regression results provide robust empirical support for Hypotheses H1, H2, H3, H4, and H5, thereby validating the proposed direct-effect relationships within the study’s theoretical framework.

### 4.3. Mediation effect test

Table 5 presents the bootstrap estimates of indirect effects, including effect sizes and confidence intervals, to assess the significance of individual mediation paths. For the mediation analysis, a bias-corrected percentile bootstrap method with 5,000 resamples was applied to estimate indirect effects and their confidence intervals. As shown in Table 5, for the mediation path DL → DSE → RAQB, the total effect (c) of digital literacy on reduced audit quality behavior was −0.278. The regression coefficient of digital literacy on digital self-efficacy (a) was 0.289, while the coefficient of digital self-efficacy on reduced audit quality behavior (b) was −0.178. The standardized indirect effect (ab) was −0.051, with a 95% bootstrap confidence interval of [−0.086, −0.024], excluding zero [41]. These results indicate a statistically significant partial mediation effect, suggesting that digital literacy influences reduced audit quality behavior both directly and indirectly through digital self-efficacy [43,44]. This indirect pathway accounted for 18.45% of the total effect, indicating that nearly one-fifth of the influence of digital literacy operates through auditors’ confidence in their digital capabilities [45].

**Table 5:** Results of mediation effect size.

Path	c	a	b	a*b	SE	95% CI	c’	PCT(%)
DL=>DSE=>RAQB	−0.278^**^	0.289^**^	−0.178^**^	−0.051	0.016	−0.086 ～ −0.024	−0.138^**^	18.452%
DL=>TP=>RAQB	−0.278^**^	0.362^**^	−0.246^**^	−0.089	0.018	−0.131 ～ −0.059	−0.138^**^	31.888%

Note: *p < 0.05 **p < 0.01; Digital Literacy = DL; Reduced Audit Quality Behavior = RAQB; Prosocial Behavior = PB; Digital Self-efficacy = DSE; Task Performance = TP

For the mediation path DL → TP → RAQB, the total effect remained −0.278. Digital literacy exhibited a significant positive effect on task performance (a = 0.362), while task performance had a significant negative effect on reduced audit quality behavior (b = −0.246). The standardized indirect effect via task performance was −0.089, with a 95% bootstrap confidence interval of [−0.131, −0.059], confirming statistical significance. This mediation effect accounted for 31.89% of the total effect, indicating that task performance represents a comparatively stronger mediating mechanism than digital self-efficacy [5]. Taken together, these findings suggest that while psychological confidence mechanisms play an important role, performance-related mechanisms constitute the dominant pathway through which digital literacy reduces audit quality-threatening behavior [43,44].

Table 6 reports the results of the chain mediation analysis, detailing the total, direct, and indirect effects of digital literacy on reduced audit quality behavior. The mediation effects were assessed using the bias-corrected bootstrap sampling method with 5,000 resamples, which is widely recommended for testing indirect effects in mediation models due to its robustness and distribution-free properties [41]. As reported in Table 6, the mediation path from digital literacy (DL) to reduced audit quality behavior (RAQB) via digital self-efficacy (DSE) was significant, as the 95% confidence interval [−0.086, −0.024] excluded zero. This result indicates that digital self-efficacy serves as a critical psychological mechanism through which digital literacy reduces audit quality-threatening behavior, consistent with social cognitive theory and prior empirical evidence [43,44].

**Table 6:** Chain mediation effect analysis.

Path	Effect	SE	t	p	95% CI	PCT(%)
Total Effect	−0.278	0.042	−6.561	0.000	−0.361 ～ −0.195	100.00%
Direct Effect	−0.137	0.042	−3.230	0.001	−0.220 ～ −0.054	49.28%
Total indirect effect	−0.141	0.021	−6.682	0.000	−0.190 ～ −0.106	50.72%
DL → DSE→RAQB	−0.052	0.016	−3.258	0.001	−0.086 ～ −0.024	18.71%
DL → TP→RAQB	−0.065	0.017	−3.832	0.000	−0.103 ～ −0.037	23.38%
DL → DSE → TP→RAQB	−0.024	0.006	−4.194	0.000	−0.037 ～ −0.015	8.63%

Note: Digital Literacy = DL; Reduced Audit Quality Behavior = RAQB; Prosocial Behavior = PB; Digital Self-efficacy = DSE; Task Performance = TP

Similarly, the mediation path DL → task performance (TP) → RAQB was statistically significant, with a 95% confidence interval of [−0.103, −0.037], providing further evidence that improved task performance mediates the relationship between digital literacy and reduced audit quality behavior. This finding aligns with previous studies demonstrating that enhanced performance efficiency and task execution reduce auditors’ propensity to engage in dysfunctional audit practices [45].

Moreover, analysis of the chain mediation path DL → DSE → TP → RAQB revealed a significant sequential indirect effect, as the 95% confidence interval [−0.037, −0.015] excluded zero. This result supports the existence of a multi-stage mechanism in which digital literacy enhances auditors’ digital self-efficacy, which in turn improves task performance and ultimately reduces audit quality-threatening behavior, consistent with recent digital work and auditing research [9,46]. The chain mediation effect accounted for 8.63% of the total effect, indicating a nontrivial contribution of this sequential mechanism [40]. Accordingly, the bootstrap results provide robust empirical support for Hypothesis H8.

### 4.4. Moderating effect test

To further examine the moderating role of prosocial behavior, interaction terms between digital literacy and prosocial behavior were introduced into the regression models. As shown in Table 4, the interaction effects were significant for reduced audit quality behavior (β = −0.251, p < 0.001), digital self-efficacy (β = 0.132, p < 0.01), and task performance (β = 0.120, p < 0.01), indicating that prosocial behavior significantly moderates the effects of digital literacy on these outcomes [42]. To clarify the nature and boundary conditions of these moderating effects, a simple slope analysis was conducted by estimating conditional effects at low (−1 SD), mean, and high (+1 SD) levels of prosocial behavior. As reported in Table 7, when prosocial behavior was low, the effect of digital literacy on reduced audit quality behavior was negative but not statistically significant (β = −0.046, p = 0.434). In contrast, this effect became significant at the mean level (β = −0.244, p < 0.001) and was strongest at high levels of prosocial behavior (β = −0.442, p < 0.001). This pattern indicates that the inhibitory role of digital literacy on reduced audit quality behavior is activated only when auditors operate within a sufficiently prosocial environment [41]. A similar strengthening trend was observed for digital self-efficacy. The positive effect of digital literacy on digital self-efficacy increased monotonically from low prosocial behavior (β = 0.143, p = 0.018) to the mean level (β = 0.264, p < 0.001) and further to high prosocial behavior (β = 0.385, p < 0.001). This finding suggests that prosocial interactions amplify the confidence-enhancing function of digital literacy by facilitating learning, support, and collaborative problem-solving [43,44].

**Table 7:** Simple slope analysis.

Variable	Levels of moderating	RC	SE	t	p	95% CI
RAQB	AVG	−0.244	0.041	−6.010	0.000	−0.324 ～ −0.165
	HIGH（+1SD）	−0.442	0.053	−8.290	0.000	−0.547 ～ −0.338
	LOW（−1SD）	−0.046	0.059	−0.783	0.434	−0.162 ～ 0.070
DSE	AVG	0.264	0.041	6.373	0.000	0.183 ～ 0.345
	HIGH（+1SD）	0.385	0.054	7.076	0.000	0.278 ～ 0.491
	LOW（−1SD）	0.143	0.060	2.375	0.018	0.025 ～ 0.262
TP	AVG	0.334	0.048	6.959	0.000	0.240 ～ 0.429
	HIGH（+1SD）	0.463	0.063	7.344	0.000	0.340 ～ 0.587
	LOW（−1SD）	0.206	0.070	2.940	0.003	0.069 ～ 0.343

Note: Digital Literacy = DL; Reduced Audit Quality Behavior = RAQB; Prosocial Behavior = PB; Digital Self-efficacy = DSE; Task Performance = TP

**Fig 2:**
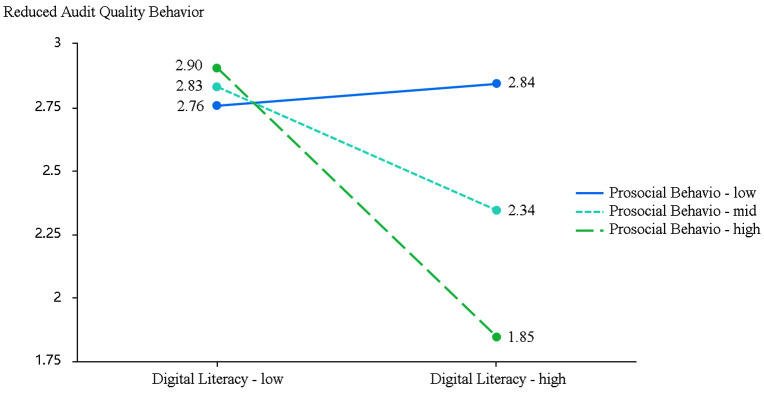
The moderating effect of prosocial behavior on digital literacy and reduced audit quality behavior.

**Fig 3:**
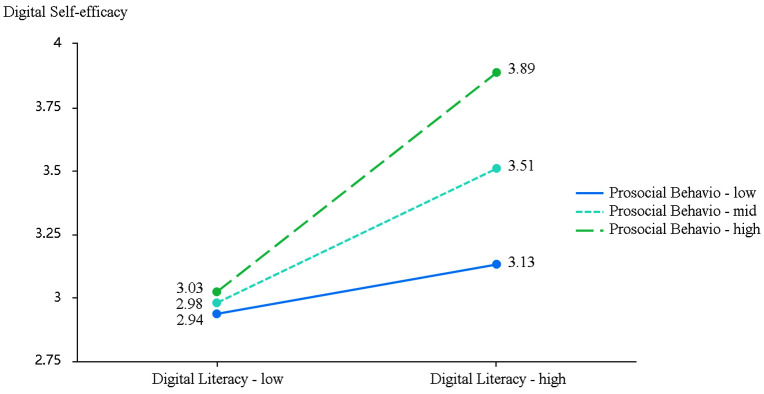
The moderating effect of prosocial behavior on digital literacy and digital self-efficacy.

**Fig 4:**
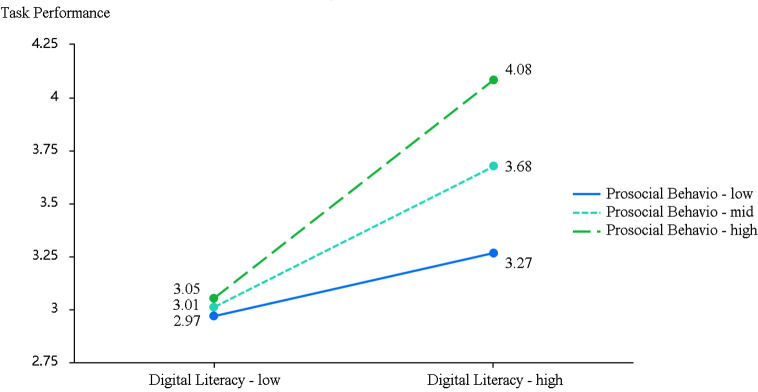
The moderating effect of prosocial behavior on digital literacy and task performance.

For task performance, the effect of digital literacy was statistically significant across all levels of prosocial behavior but increased substantially as prosocial behavior rose (β = 0.206, p = 0.003; β = 0.334, p < 0.001; β = 0.463, p < 0.001). This indicates that while digital literacy directly improves task execution, a prosocial context magnifies its effectiveness by enabling better coordination and resource sharing [9,46]. These results demonstrate that prosocial behavior establishes clear boundary conditions for the effectiveness of digital literacy [41]. When prosocial behavior is weak, digital skills alone are insufficient to curb reduced audit quality behavior [43,44]. As prosocial behavior increases, however, digital literacy becomes significantly more effective in enhancing auditors’ self-efficacy and task performance and, in turn, suppressing audit quality-threatening behavior [41]. Accordingly, Hypotheses H9, H10, and H11 are supported. The results of the simple slope analysis are illustrated in Figs 2–4. Figs 2–4 present interaction plots illustrating the moderating role of prosocial behavior at high (+1 SD) and low (−1 SD) levels on the relationships between digital literacy and reduced audit quality behavior, digital self-efficacy, and task performance, respectively. These figures clearly show that the slopes representing the relationship between digital literacy and the outcome variables become steeper under higher levels of prosocial behavior, indicating that the effects of digital literacy are substantially stronger in more prosocial work environments. The graphical patterns are consistent with the results of the simple slope analysis reported in Table 7 and further illustrate the boundary conditions under which digital literacy exerts stronger behavioral and performance-related effects.

To help visualize the different effect sizes of digital literacy for different prosocial behavior, β coefficients of regression analyses were used as slopes of each line. For high-prosocial behavior, digital literacy’s effect on reduced audit quality behavior was of β2.90; middle-prosocial behavior’s β was 2.83; and low-prosocial behavior’s β was 2.76.

To help visualize the different effect sizes of digital literacy for different prosocial behavior, β coefficients of regression analyses were used as slopes of each line. For high-prosocial behavior, digital literacy’s effect on digital self-efficacy was of β3.03; middle-prosocial behavior’s β was 2.98; and low-prosocial behavior’s β was 2.94.

To help visualize the different effect sizes of digital literacy for different prosocial behavior, β coefficients of regression analyses were used as slopes of each line. For high-prosocial behavior, digital literacy’s effect on task performance was of β3.05; middle-prosocial behavior’s β was 3.01; and low-prosocial behavior’s β was 2.97.

### 4.5. Test of moderated mediation effect

To further examine whether prosocial behavior conditions the indirect effects of digital literacy on reduced audit quality behavior, a moderated mediation analysis was conducted [43,44]. The results are presented in Table 8. The analysis employs the index of moderated mediation, which captures whether and to what extent the magnitude of an indirect effect varies systematically across levels of the moderator, rather than merely testing its overall significance.

**Table 8:** Index of moderated mediation.

Moderator Variables	Mediating Variables	Index	SE	95% CI
PB	DSE	−0.033	0.013	−0.060 ～ −0.012
	TP	−0.045	0.019	−0.087 ～ −0.010

Note: Digital Literacy = DL; Reduced Audit Quality Behavior = RAQB; Prosocial Behavior = PB; Digital Self-efficacy = DSE; Task Performance = TP

For the mediation pathway involving digital self-efficacy, the index of moderated mediation was −0.033, with a bootstrap confidence interval of [−0.060, −0.012], excluding zero. This result indicates that prosocial behavior significantly moderates the indirect effect of digital literacy on reduced audit quality behavior via digital self-efficacy [9 46]. Specifically, as prosocial behavior increases, the negative indirect effect becomes stronger, suggesting that a supportive and cooperative social environment enhances the extent to which digital literacy translates into greater self-efficacy and, consequently, lower audit quality-threatening behavior.

Similarly, for the mediation pathway through task performance, the index of moderated mediation was −0.045, with a bootstrap confidence interval of [−0.087, −0.010]. This finding demonstrates that prosocial behavior also significantly moderates the indirect effect of digital literacy via task performance [5]. Higher levels of prosocial behavior amplify the positive influence of digital literacy on task execution, thereby strengthening its indirect inhibitory effect on reduced audit quality behavior.

Taken together, these results indicate that prosocial behavior not only moderates the direct relationships between digital literacy and key outcomes but also reshapes the internal transmission mechanisms through which digital literacy influences audit quality. In line with social cognitive theory, a prosocial work environment enhances learning, confidence building, and cooperative task execution, thereby facilitating the effective conversion of individual digital capabilities into high-quality audit behavior [41].

From a practical perspective, the negative values of the moderated mediation indices imply that fostering prosocial behavior can significantly enhance the governance role of digital literacy. When auditors operate in a cooperative and supportive environment, investments in digital skills are more likely to yield substantial improvements in task performance and reductions in audit quality-threatening behavior [45]. Accordingly, Hypotheses H12 and H13 are supported.

### 4.6. Robustness of empirical results

To further assess the robustness of the empirical findings, multiple complementary analytical techniques were employed throughout the empirical analysis. Direct, mediating, and moderating effects were estimated using hierarchical regression models, while mediation, chain mediation, and moderated mediation effects were tested using a nonparametric bootstrap procedure with 5,000 resamples [41]. Bootstrapping does not rely on normality assumptions and is widely recommended for assessing indirect effects, thereby enhancing the reliability of statistical inference [5]. The consistency and stability of indirect effects were evaluated through repeated bootstrap estimation, with all confidence intervals excluding zero, indicating that the mediation and chain mediation effects were statistically robust. In addition, interaction effects between digital literacy and prosocial behavior remained statistically significant across alternative model specifications, supporting the robustness of the moderating relationships. Collectively, the results demonstrate that the hypothesized relationships are stable, internally consistent, and not sensitive to estimation approach or model specification [5]. These robustness checks provide additional assurance that the empirical conclusions drawn in this study are reliable.

## 5. Conclusion and implications

### 5.1. Research conclusions

Driven by digital transformation in auditing, this study examined how auditors’ digital literacy influences reduced audit quality behavior through psychological and behavioral mechanisms. The findings indicate that digital literacy plays a critical role in improving audit quality by reducing audit-quality-threatening behavior, primarily through enhancing auditors’ internal cognitive resources and work-related capabilities.

More importantly, the results demonstrate that the influence of digital literacy is not limited to a direct effect but operates through a structured psychological–behavioral process. Digital self-efficacy and task performance function as key transmission mechanisms, suggesting that digital competence first strengthens auditors’ confidence in using digital tools, which subsequently improves their execution of audit tasks. This sequential pathway provides a more integrated explanation of how digital capabilities translate into improved audit behavior.

In addition, prosocial behavior emerges as an important boundary condition that strengthens the effectiveness of digital literacy. A supportive and cooperative work environment enhances the extent to which digital literacy is converted into self-efficacy and performance improvement. Overall, the findings suggest that audit quality improvement is jointly shaped by technological capability, psychological confidence, task execution, and social context.

### 5.2. Theoretical contributions

This study contributes to the literature on auditing and digital competence in three main ways.

First, it reinforces and extends human capital theory by demonstrating that digital literacy is a critical form of job-relevant human capital that reduces dysfunctional audit behavior. Rather than treating digital literacy as a purely technical skill, the findings highlight its behavioral consequences in shaping audit quality outcomes in digitally intensive environments.

Second, this study advances self-efficacy theory by identifying a sequential mechanism linking digital literacy, digital self-efficacy, and task performance. Previous research has typically examined these constructs in isolation; this study integrates them into a coherent chain mediation model, offering a more comprehensive explanation of how digital capability translates into behavioral outcomes.

Third, by introducing prosocial behavior as a moderating factor, this study extends existing audit behavior research beyond individual-level explanations. The results highlight that audit outcomes are not determined solely by technical competence or psychological factors, but are also conditioned by the surrounding social environment. This provides a more holistic understanding of audit quality formation in digital contexts.

### 5.3. Managerial implications

The findings offer several practical implications for audit firms, regulators, and professional bodies.

First, improving digital literacy should be treated as a structured capability-building process rather than a uniform training requirement. Training programs should be differentiated according to auditors’ roles, with foundational digital skills emphasized for junior auditors and advanced analytics and AI-based judgment tools targeted at senior staff.

Second, strengthening digital self-efficacy is essential for translating digital skills into behavioral outcomes. Audit teams should create structured learning environments that include progressive task assignments, real-time feedback, and mentoring systems to enhance auditors’ confidence in applying digital tools.

Third, task performance represents the most influential transmission pathway in reducing audit-quality-threatening behavior. Therefore, firms should focus on optimizing audit workflows, integrating digital tools into core audit procedures, and reducing unnecessary manual workload to improve efficiency and decision accuracy.

Fourth, fostering prosocial behavior within audit teams can significantly enhance the effectiveness of digital capability development. Encouraging knowledge sharing, collaboration, and peer support can strengthen both confidence and performance outcomes in digital auditing environments.

Finally, regulators and professional bodies should incorporate digital literacy and related behavioral competencies into audit quality assessment and continuing professional development systems to ensure consistent capability standards across the profession.

### 5.4. Limitations and future research directions

This study has several limitations that suggest directions for future research.

First, the cross-sectional design limits causal inference. Although theoretical reasoning supports the proposed relationships, longitudinal or experimental designs would provide stronger evidence regarding temporal and causal ordering, particularly between digital literacy and audit behavior.

Second, the reliance on self-reported data may introduce common method bias and social desirability effects. Future studies should incorporate multi-source data, such as supervisor evaluations, audit records, or behavioral trace data, to improve measurement validity.

Third, the sample is derived from a specific institutional context, which may limit generalizability. Future research should test the model across different regulatory environments and cultural settings to enhance external validity.

Finally, future studies could further explore contextual and multilevel mechanisms, such as organizational culture, technological infrastructure, or team dynamics, to better understand how digital transformation influences audit behavior in practice.

## Supporting information

S1 File Dataset. (XLSX)
